# Determination of Elemental Composition of *Malabar spinach*, Lettuce, Spinach, Hyacinth Bean, and Cauliflower Vegetables Using Proton Induced X-Ray Emission Technique at Savar Subdistrict in Bangladesh

**DOI:** 10.1155/2015/128256

**Published:** 2015-07-02

**Authors:** S. M. Fahad, A. F. M. Mahmudul Islam, Mahiuddin Ahmed, Nizam Uddin, Md. Rezaul Alam, Md. Ferdous Alam, Md. Farhan Khalik, Md. Sazzad Hossain, Md. Lokman Hossain, Md. Joynal Abedin

**Affiliations:** ^1^Department of Physics, Jahangirnagar University, Savar, Dhaka 1342, Bangladesh; ^2^Department of Pharmacy, Jahangirnagar University, Savar, Dhaka 1342, Bangladesh; ^3^Department of Pharmacy, Gono Bishwabidyalay, Mirzanagar, Savar, Dhaka 1344, Bangladesh; ^4^Pharmacy Discipline, Khulna University, Khulna 9208, Bangladesh; ^5^Accelerator Facilities Division, Atomic Energy Centre, Dhaka (AECD), Bangladesh

## Abstract

The concentrations of 18 different elements (K, Ca, Fe, Cl, P, Zn, S, Mn, Ti, Cr, Rb, Co, Br, Sr, Ru, Si, Ni, and Cu) were analyzed in five selected vegetables through Proton Induced X-ray Emission (PIXE) technique. The objective of this study was to provide updated information on concentrations of elements in vegetables available in the local markets at Savar subdistrict in Bangladesh. These elements were found in varying concentrations in the studied vegetables. The results also indicated that P, Cl, K, Ca, Mn, Fe, and Zn were found in all vegetables. Overall, K and Ca exhibited the highest concentrations. Cu and Ni exhibited the lowest concentrations in vegetables. The necessity of these elements was also evaluated, based on the established limits of regulatory standards. The findings of this study suggest that the consumption of these vegetables is not completely free of health risks.

## 1. Introduction

Minerals containing food plants are of prime concern as these plants contain the prime resource of essential elements (without hydrogen and oxygen) in the human nutrition [1]. A little quantity of these minerals originates from soil, water, air, and rocks. The elements which are confined in the plants are obtained directly through fruits and vegetables, or indirectly through milk and meat from those animals that consume the plants. Trace elements do not provide any calorie to the human body but play an important role in the adjustment of the body pH, osmotic regularity and are used as coenzyme which regulates the elemental metabolic processes in the human cell. In recent years trace elements have become essential to the biological cell cycling and homeostasis process [2, 3]. In the human body excessive accumulation of potentially toxic elements or lack of essential trace elements for a long period is responsible for various diseases [4]. Improvement of the mineral regulation, reduction of cardiovascular diseases, and certain cancer risks can be achieved through the increase in the consumption of various fruits and vegetables. The levels of trace elements in fruits and vegetables may be regulated by various factors like genetic, soil chemistry and the pattern of agricultural system [5]. The edible components of the plants like leaves and stems and the concentrations and the types of elements available in these parts are greatly influenced by atmospheric contamination. If tubers and roots are consumed as food, the elements that are found in the soil may reveal the maximum effect on the levels of minerals of fruits and vegetables, whereas elements confined in fruits are prone to be significantly influenced by the variations in the geochemical settings. Minerals can be divided into three groups: major physiological elements, trace elements, and nonessential elements [6]. Major physiological elements are phosphate, bicarbonate, Na, K, Ca, Mg, Cl, and so forth. Essential and trace elements are Co, Cr, Cu, Fe, I, Mn, Mo, Ni, S, Se, Zn, and so forth. Nonessential elements like As, Al, Br, Ba, Bi, Cs, Cd, Ge, Hg, Li, Pb, Rb, Sr, Si, Sm, Sb, Sn, Ti, Tl, and W are not currently considered essential, even though some of them have a beneficial pharmacological action in appropriate dosage [6]. The nonnutritive toxic minerals which are commonly known to have toxic properties even in small quantities (<100 ppm) are Pb, Sb, Cd, As, Se, and Hg [7]. For this reason, the estimation of both physiological and trace levels of elements obtained in food are significant for safety, efficacy, and nutritional values of food [4]. The aim of this study was the precise determination of the contents of minerals and heavy elements from the most known and available vegetables present in Bangladeshi market.

Before selecting these vegetable samples, at first short discourse was performed with the vegetable vendors in some local markets at Savar. Vegetable vendors were asked to suggest some vegetables which are sold most. They provided some popular names of vegetables. Most of the people in Savar area eat these vegetables to receive nourishment and for the availability of these vegetables at affordable cost. This information proves that most important vegetables, which are mostly consumed by the people living in Savar area, were selected to perform this study. Consumers living in Savar area usually welcome these types of foodstuffs into their regimens. These foods are cheap and contain low fat. Moreover, they are very tasty, easy to find in the grocery store, and also suitable to cook. Both cooked and raw vegetables are very rich sources of different types of minerals and vitamins. After that, high nutrition values of these selected vegetables were ensured by studying different literatures. From the preliminary selected vegetables, finally the top 5 vegetables were selected for our research. Collection of the samples was performed several times a week from nearby markets in Savar area.

## 2. Materials and Methods

### 2.1. Sample Preparation

Five different vegetables* Basella alba* (*Malabar spinach*),* Lactuca sativa *(lettuce),* Spinacia oleracea* (spinach),* Lablab niger* (hyacinth bean), and* Brassica oleracea* (cauliflower) were collected from different places of Savar area, Bangladesh. For each category of sample, we collected 10 vegetable samples. All determinations of essential and trace elements in collected vegetables were performed by the technique Proton Induced X-ray Emission (PIXE). The technique PIXE is being used in Accelerator Facilities (AF) Division of the Atomic Energy Centre, Dhaka (AECD), Bangladesh. The fundamental benefit of the PIXE method is to determine the concentrations (absolute or relative) of elemental constituents in the sample under investigation. The collected samples were washed thoroughly with distilled water and then dried in an oven at 80°C and consequently grinded into fine powder through using mortar and pestle. Then pellets having a diameter of 12 mm were made from powder of samples by using a 15 ton Perkin Elmer pellet maker.

### 2.2. PIXE Technique

Proton Induced X-ray Emission (PIXE) is one of the important and widely used analytical techniques at MeV energy accelerators. Sven Johansson at the department of Physics, Lund University, first introduced this technique [8]. When charged particles with sufficient energy hit a sample, a vacancy in the inner shells of an atom may be created. For MeV ions the probability (cross section) for ejecting inner shell electrons is very high [8]. Such a vacancy can be occupied in a number of techniques and one of the processes may discharge X-rays with energy characteristic of that specific atomic number. In the PIXE technique X-rays are detected using semiconductors. An energy dispersive analysis of the detector signals can disclose the distinctiveness of different elements existing in the samples analyzed and more notably by quantifying the charge; that is, the number of incoming elements and the concentrations of the elements can be precisely quantified. PIXE technique has excellent detection limits, so the concentrations of the elements can be accurately quantified [9]. This technique was chosen because of its advantages. It is a nondestructive, very sensitive, and multielemental technique which permits rapid and simultaneous detection of elements between Na and U [10, 11]. PIXE technique is the reliable measurement of toxic elements in the bioenvironmental samples in parts per million (ppm) levels and also supports detecting the presence of any essential elements. It also buttresses in making decision about conservation of the public health and improving environmental quality [8].

### 2.3. Instrumentation

#### 2.3.1. Experimental Setup

The elemental analysis was carried out at Accelerator Facilities (AF) Division of the Atomic Energy Centre, Dhaka (AECD), Bangladesh. The prepared pellet samples were irradiated at the PIXE station for elemental analysis with 2.5 MeV collimated proton beam (size approximately of 2 mm) at a current of about 10 nA. The experimental samples were irradiated with approximately 10 *μ*C charge and it was obtained from the 3 MeV Van de Graff Accelerator. All experiments were done in the vacuum chamber. The vacuum in the range of the order of 2.0 × 10^−5^ mbar is regularly achieved in the chamber. The turbo pumping system is used to evacuate the accelerator tube and rest of the beam line. A small turbo pump is being used with the IBA scattering chamber for vacuum purpose. Characteristic X-rays from targets were measured with a Si(Li) detector with respect to the beam line. Detector was connected to a multichannel analyser (MCA) in order to convert analogue data into digital. 170 *μ*m thick Mylar absorber was used in front of the X-ray detector for two reasons. Firstly, to save the X-ray detector from radiation damage from the scattered high energy particles from the sample and secondly to decrease the count rate in the X-ray detector which is very important in order to decrease pulse pile up and the dead time in the data-acquisition system [8]. Accurate charge measurement is very essential for the complete quantification of elemental concentrations of a sample [8]. The most imperative factors that were considered during charge measurements were loss of secondary electrons, loss of scattered particles, and charge build up in insulating samples. An electron flood gun was used in all experiments in order to prevent charge build up in the samples [12]. The charge measurement was actually done in the Faraday cup which was positioned at the end of the beam line after the chamber. The low *Z* elements present in the sample that produce most of the counts were compared to the tracer level of high *Z* elements in the sample.

#### 2.3.2. PIXE Calibration

GUPIX was chosen to analyse complex PIXE spectra of sample from a number of software packages available in this field. It was developed in the Guelph University, Canada. GUPIX uses all the modernized and best databases available like cross sections, fluorescence, stopping powers, Coster-Kronig probabilities and attenuation coefficients. It has many convenient options such as: the possibility to add invisible elements to the fit, matrix iteration, layer thickness iteration, analysis of any batch spectra, and so forth.

It also possesses the possibility to include an energy-dependent calibration factor *H* and low energy tailing descriptions of parameter of the X-ray detector in the database [13]. All of these measurements greatly improve the accuracy of the quantification of elemental concentrations of samples through using PIXE technique. Mainly the *H* value method was followed during the PIXE calibration process [14, 15] that is based upon the equation given below:

(1) $$\begin{array}{c} Y\left(\;Z,M\;\right)=Y_{I}\left(\;Z,M\;\right)\cdot Q\cdot C_{Z}\cdot T\left(\;Z\;\right)\cdot \varepsilon_{Z}\cdot H, \end{array}$$

where *Y*(*Z*, *M*) is the measured X-ray yield computed by the fitting program. *Y*_*l*_(*Z*, *M*) is the theoretical X-ray yield per unit beam charge, per unit solid angle, and per unit concentration computed from the GUPIX database which includes the matrix correction and secondary fluorescence for the thick targets. *Q* is the measured beam charge or some value proportional to the charge. *C*_*Z*_ is the concentration of the element quoted by the manufacturer or measured by some other method. *T*(*Z*) is the fractional transmission of X-rays through absorbers. *ε*_*Z*_ is the intrinsic detector efficiency. *H* is the product of the detector solid angle and any correction factor for the charge measurement.

So in this case, *H* also includes the effects of possible inaccuracy in the description of the detector solid angle, the thickness of the detector crystal, the thickness of the absorber layer, and so forth. It also includes any imperfection in the various databases used in GUPIX [16, 17]. The energy-dependent *H*-values and low energy tailing parameters are detector specific [13]. To evaluate the *H*-values a whole set of thin Micromatter standard was irradiated. First, by using the actual solid angle as the *H* value in the GUPIX the concentration values were calculated and then the thickness of the absorber was adjusted to get the closest concentration value, the manufacture quoted value, especially for those elements whose X-ray was sensitive to absorb that particular thickness. Then *H* values were calculated by comparing the calculated concentration values to the manufacturer quoted concentration values multiplied by the detector solid angle [13]. The *H* values were then plotted as function of X-ray energy, which was a scattered curve, and fitted by a suitable trend line to obtain an equation. Then this equation was used to generate a smooth set of *H* values as function of X-ray, which was stored inside the GUPIX library for further PIXE analysis of unknown samples. These *H* values were used as function of energy and this was suggested by GUPIX. Depending on the element, the precision was well below 1% and the accuracy was within 5–10%.

An excellent procedure has been established by Kim et al. [18] for determining the elemental composition of any biological sample. The limit of detection (LOD) meant the sensitivity of any measurement system. Limit of detection is clearly proportional to the solid angle. In PIXE analysis, detection limits are usually calculated by the following equation:

(2) $$\begin{array}{c} \text{Limit of detection}=3\cdot \frac{\sqrt{BG}}{S}\cdot C. \end{array}$$

### 2.4. Data Analysis

#### 2.4.1. PIXE Standardization Procedure

Calibration and analytical methodologies of the PIXE technique were performed by analyzing appropriate reference material called IAEA-V-10 (Hay powder). The concentrations of the standard were also prepared in a similar manner with the studied vegetable samples. In order to validate our results, the elemental concentrations of standard samples were measured several times in the same experimental conditions of the analyzed vegetable samples. Relative standard deviations are mostly within ±5–10%, which may recommend good precision.

#### 2.4.2. Data-Acquisition System

The data-acquisition system is one of the important parts of an ion beam experimental setup like PIXE technique [19]. MAESTRO-32 (Ortec, USA, version 6.05) interface software was used for spectrum data acquisition. X-ray spectra of vegetable samples were analyzed using GUPIX. GUPIX software with the help of DAN32 interfacing software has become convenient to analyze any complex PIXE spectra [20].

## 3. Results and Discussion

In case of reference material IAEA-V-10 (Hay powder), the deviations of the measured values were mostly within ±5–10% which may represent a good agreement between the measured and certified values (Table 1).

Table 2 shows a wide variation in the elemental concentrations of the different vegetable samples studied. Several trace elements are important micronutrient to the human body and are required for wellbeing of body immune system. The elements that were found in this study are silicon, phosphorus, chlorine, potassium, calcium, titanium, manganese, iron, cobalt, zinc, strontium, bromine, rubidium, chromium, ruthenium, nickel, sulfur, and copper (Table 2). The concentration level of each element in the selected vegetables was found in the decreasing order as K > Ca > Fe > P > Cl > Zn > S > Mn > Ti > Co > Br > Cr > Si > Ru > Rb > Sr > Cu > Ni (Table 2).

**Figure 1:**
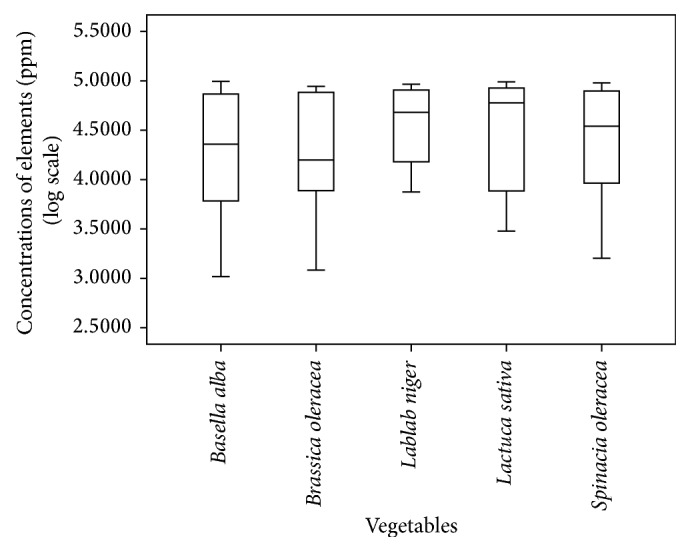
Distribution of 18 elements in five different vegetable samples is shown in box plot.

The distributions (in log scale) of bromine, rubidium, phosphorus, sulfur, strontium, ruthenium, chlorine, potassium, chromium, manganese, iron, cobalt, copper, calcium, titanium, zinc, nickel, and silicon within five different vegetables are shown using a side-by-side box plot (Figure 1).

Calcium contents in five vegetables varied in wide range of 98652–80743 ppm. The highest concentration of Ca was found in* Basella alba* (98652 ppm) and the lowest concentration was found in* Brassica oleracea* (80743 ppm). Ca is important due to the dietary significance of such cations and is the 5th most common element found in the human body. It is an obvious structural material, being present in cell walls, bones, and teeth. Calcium intakes protect against the risk of hypertension and decrease bone loss and the risk of kidney stones [21]. Calcium initiates changes in protein shape through binding to thousands of cell proteins. Thus it governs cellular functions like muscle contraction and cell movement to nerve transmission, glandular secretion, and cell division [22]. Adequate intakes (AI) levels of Ca for infant from birth to 6 months and from 6 months to 1 year are 210 mg/day and 270 mg/day, respectively. The RDAs of Ca for these same age groups are 200 and 260 mg/day, respectively. AI levels of Ca for children of 1–5 years and 4–8 years are 500 and 800 mg/day, respectively, and RDAs of Ca for these same age groups are 700 and 1000 mg/day, respectively. AI and RDA levels of Ca for adolescents of 8–18 years, adults of 19–50 years, and >50 years are 1300, 1000, and 1200 mg/day, respectively. AI and RDA levels of Ca for pregnant and lactating women of 14–18 years and >19 years are 1300 and 1000 mg/day [23, 24]. Mean calcium intakes will be higher than the recommended intake for calcium in persons dependent on these types of vegetables. Thus people of these areas are at high risks of excess dietary calcium. Pregnant women and elderly persons are vulnerable to the development of some diseases like hypercalcemia, metabolic alkalosis, and possibility of renal insufficiency. It has revealed that calcium may protect against the development of colon and breast cancer [25, 26]. Cancer patients may be relieved from chemotherapy and radiation therapy induced osteopenia and osteoporosis due to proper calcium intake. Some of these vegetables also provide protection against cancer and osteoporosis that is correlated well with significant concentrations of calcium present in them.

The elemental concentration of potassium in* Lactuca sativa* vegetable was 97723 ppm, which was the highest amount of potassium among various samples studied. The lowest amount of potassium was exhibited by* Brassica oleracea* (83412 ppm). The elemental concentrations of potassium of* Basella alba, Spinacia oleracea,* and* Lablab niger* were 91632 ppm, 88021 ppm, and 92381 ppm, respectively. An increased level of potassium seems to augment the necessity of sodium and vice versa. The high level of potassium in these types of vegetables analyzed supports the idea that these plants may have high absorption rate of trace heavy metals from the soil where it grows and there may be plenty of potassium available in the soil of those vegetable plots. Potassium plays a notable role in maintaining the human's body fluid balance. Potassium is the primary cation inside cells; notably all of these vegetables are rich sources for potassium and calcium. So these vegetables can be eaten as DASH (Dietary Approaches to Stop Hypertension) diet, which also helps to reduce blood pressure. Potassium insufficiency directs to an enhancement of the basic amino acid amount of the tissue fluids and some raise of cellular sodium levels as a means of sustaining anion-cation balance. Potassium deficiency affects the collecting tubules of the kidney, ultimately causing the incapability of concentrate urine. It is also responsible for the alterations of intestinal motility and gastric secretions [27]. 1875–5625 mg/day is the Recommended Dietary Allowances (RDAs) of potassium for adults and children above the age of 4 years [6]. Elemental concentrations of K in five different vegetables are fortunately above the RDA value and can serve as rich sources of K.

Only one vegetable sample, namely,* Lactuca sativa,* had concentrations of Ru at a level of approximately 6301 ppm. Ruthenium has no recognized therapeutic importance to the human body [28]. Ru has the propensity to be mobile in the soils and thus can be easily taken up and gathered in various plant species.

The concentration of Rb was detected in both* Spinacia oleracea* (1595 ppm) and* Lactuca sativa* (4426 ppm). Rubidium is normally observed in animal tissue and it is similar to the potassium during its delivery and excretory pattern [29]. The inclusion of rubidium to potassium-deficient diets helps to avert the lesions characteristic of potassium reduction in rats [30]. It was observed that the aqueous extract of* Spinacia oleracea* eaves has protective effect, for example, attenuating the spread of lesions [31]. The presence of Rb in* Spinacia oleracea* substantiates the therapeutic usage of it against the development of lesions. From reviewing of different literatures on* Lactuca sativa*, it can be postulated that the presence of Rb was not correlated with its potential healing effects. So, further investigation is needed to understand the correlation.

Nickel was detected in only one sample named* Brassica oleracea* and the amount was 1214 ppm. Most of the nickel in food remains unabsorbed by the gastrointestinal tract and usually less than 10% of nickel ingested with food is absorbed [32]. The maximum permissible concentration of Ni in vegetables and fruits is 0.5 mg/kg [33]. In the present study the value of Ni observed in* Brassica oleracea* actually violated the permissible limit of Ni consumption as set by International Programme on Chemical Safety (IPCS) in its Health and Safety Guide number 62. Ni is well known as an activator of a number of enzymes, for example, alkaline phosphatase and oxaloacetate decarboxylase. So taking of* Brassica oleracea* (cauliflower) along with diet may be advantageous for the activation of these enzymes.

The concentration of Mn differed markedly among vegetable samples. The highest Mn content (59861 ppm) was found in* Lactuca sativa* and the lowest value (1852 ppm) was determined in* Basella alba*. The concentrations of Mn found in* Spinacia oleracea*,* Lablab niger,* and* Brassica oleracea* were 13222, 39402, and 9018 ppm, respectively. Manganese functions as a cofactor of decarboxylase, transferase, and hydrolase enzymes in human body. In this sense, the determination of trace elements like Mn in food is of great importance since the deficiency or excess of Mn could promote several clinical disorders resulting in serious health problems. Deficiency of Mn in human body is the prime cause of myocardial infarction and other cardiovascular diseases. Mn deficiency is also characterized by CNS malfunctions, bone abnormalities, defective growth, reproductive dysfunction and disturbances in lipid and fat metabolism, and so forth [34]. The safe and sufficient level of daily manganese recommended intake range from 1.2 to 1.5 mg/day for children up to 8 years, 1.9–2.3 mg/day for male up to 70 years, and 1.6–1.8 mg/day for female up to 70 years [35]. However, it is essential to note that the levels of Mn in vegetable samples were very high for these aged groups. Manganese appears to be the ideal metal for oxidative stress protection. Protection of Mn against radiation, oxidative stress, and backup for SOD (Super Oxide Dismutase) enzymes were collectively referred to as manganese antioxidant [36]. The presence of Mn in all five vegetable samples analyzed also substantiates that these vegetables may give protection against radiation and oxidative damage.

Phosphorus is a ubiquitous mineral and acts as an integral component of the body's vital energy source, ATP. Phosphorus-containing compounds such as cAMP, cGMP, and IP3 regulate many intracellular signaling processes in the body [37].* Brassica oleracea* had the highest phosphorus concentration (87856 ppm) followed by* Lactuca sativa* (78342 ppm). The lowest phosphorous concentration (14314 ppm) was showed by* Lablab niger*. Both* Basella alba* and* Spinacia oleracea* showed phosphorus concentration of about 22827 and 65105 ppm, respectively. The recommended dietary allowances of phosphorous for children of 1–3 years, for children of 4–8 years, for males/females of 9–18 years, for males/females of 19–70 years, and also for males/females of >70 years are 460 mg/day, 500 mg/day, 1250 mg/day, 700 mg/day, and 700 mg/day, respectively [23]. The values observed in five different vegetables were above these dietary allowances proposed by Food and Nutrition Board. These vegetables can be used as a staple food source for phosphorous. These are also useful for the primary prevention of osteoporosis.

The biological role of titanium is not fully understandable and has been studied for more than 90 years [38]. There is no strong evidence of a biochemical role of titanium. Some plants depend on Ti to perform photooxidation of nitrogen compounds and some processes of photosynthesis [39]. The presence of Ti in two of vegetable samples may be due to commencement of these biochemical pathways. The concentration of titanium was high in* Spinacia oleracea* (33682 ppm) whereas low in* Basella alba* (24026 ppm). High concentrations of titanium can possibly be found in foods or vegetables available in local market as a result of soil contamination by fly ash fallout [40–42], use of industrial sewage, and household waste. Titanium has no well-known biological use in human body, although it is known to act as a stimulating agent. No adequate intakes (AI) or recommended dietary allowances (RDAs) have been determined for Ni. A study was conducted in 1969 by Asmaeva and Il'vickij revealed that levels of titanium in foods were affected by climatic conditions and the region of growth [43].

Micromineral copper content was found to be nil in four vegetables except* Brassica oleracea* that showed an amount of 1264 ppm. Copper deficiency in human may lead to hypochromic, microcytic anemia, neutropenia, cardiovascular system abnormalities, bone and cartilage instability, hypopigmentation, and abnormal keratinization of hair [37, 44]. Two copper containing superoxide dismutase enzymes protect human body against free radicals. Cu also helps to heal any type of wound. Adequate intake (AI) levels for copper have been recognized for infants of 0 to 6 months of age and for those between 7 and 12 months as 200 and 220 *μ*g/day, respectively. The RDAs for 1–3 years, 4–8 years, 9–13 years, 14–18 years, and 19–50+ years of age are 340, 440, 700, 890, 900, and 900 mg/day, respectively. The RDAs during pregnancy (14 through 18 years and 19 through 50 years) and lactation (14 through 18 years and 19 through 50 years) are 1000 and 1300 *μ*g/day, respectively [45]. Unfortunately Cu concentration observed in* Brassica oleracea* was higher than allowance. Although copper is indispensable to the good health but excessive consumption may result in serious health problems like kidney and liver damage [46]. Because of these possible adverse consequences from high copper ingestion, an endurable upper intake level (UL) of 10 mg/day has been established for adults who are older than 19 years of age. According to the results of this study conducted in five vegetable samples,* Brassica oleracea* is a highly rich vegetable for its Cu content. Thus this vegetable may be associated with some potential healing effects on free radicals and also may protect humans from developing cardiovascular diseases.

Zinc is an indispensable or essential element for animals [47] and almost 300 mammalian enzymes are related to Zn [37]. The results indicated that all vegetable samples contained Zn. The amount of Zn was in the range of 14022–82467 ppm. Zn concentration was considerably higher in* Lactuca sativa* (82467 ppm) than in rest of the four different vegetable samples, for example,* Basella alba*,* Spinacia oleracea*,* Lablab niger,* and* Brassica oleracea*. Previous studies revealed that optimal level of Zn is required for the human body to reduce morbidity resulting from diarrheal and respiratory diseases [48]. Additionally, Zn deficiency can also cause retardation of growth and development, immune system deficiencies (i.e., lymphopenia, thymic defects, reduced phagocytosis, depressed T-cell function, and impaired cytokine production), behavioral disturbances (including impaired hedonic tone), impaired taste (hypogeusia), delayed healing of wounds and burns, decubitus ulcers, and cognitive function impairment [37]. The RDAs of Zn per day are 4 mg for children of about 4–8 years, 8 mg for children of about 9–13 years, 11 mg for male children of about 14–18 years, 11 mg for male adults of about 19–71+ years, 9 mg for female children of about 14–18 years, and 8 mg for female adults of about 19–71+ years of age [35]. The observed concentration of Zn in vegetables strengthens its abundance. It was observed that there is a direct association between zinc deficiency and cancer [49–51]. Zinc has played protective role against carcinogenesis and the presence of considerable amounts of zinc in all these vegetable samples just buttresses their usage for the treatment of cancer.

An interesting observation made in this work is the absence of chromium in* Basella alba*,* Spinacia oleracea*,* Lablab niger*, and so forth. The amounts of chromium in* Lactuca sativa* and* Brassica oleracea* were approximately 9335 and 9112 ppm, respectively. These considerable levels were attributed to external sources such as processing, preparation, and storage. Chromium is another essential trace element and is necessary for optimal growth. Chromium has been shown to normalize the impaired glucose tolerance of some diabetes, old patients, and malnourished children [6]. 50–200 *μ*g/day is the RDA of Cr for adults and children above 4 years [52]. This highest level of Cr in* Brassica oleracea* and* Lactuca sativa* may be influenced by the local soil and water conditions of the vegetable plots. Vegetable plants do not need Cr and normally contain <0.2 mg/kg but plants could bioaccumulate Cr from soil and water. When different types of vegetable plants are grown in soil polluted from Cr-emitting industries or when sewage sludge is used as a fertilizer, these plants can accumulate high concentrations of chromium [53]. An upper tolerable limit or UL value for Cr has not yet been established. Cr has played a vital role in the metabolism of cholesterol and in-heart diseases [54]. The presence of considerable amounts of Cr in* Lactuca sativa* and* Brassica oleracea* may be correlated with their therapeutic properties against diabetes mellitus and cardiovascular diseases.

Within the selected vegetables, the highest concentrations of Fe were noticed in* Lactuca sativa* (90525 ppm) followed by* Spinacia oleracea* (83411 ppm),* Lablab niger* (80761 ppm),* Basella alba* (72325 ppm), and* Brassica oleracea* (72123 ppm) in decreasing concentrations. The U.S. Food and Nutrition Board (FNB) has already assessed that the iron bioavailability from the diet of a vegetarian is only 10%, while mixed diet has iron bioavailability of 18%. Therefore, the RDAs for iron are of 10 mg/day for children of 4–8 years, 11 mg/day for males of 14–18 years, 15 mg/day for females of 14–18 years, 8 mg/day for 19–50 years of male adults, 18 mg/day for 19–50 years of female adults, and 8 mg/day for >50 years of male/female adults [35]. In the least-developed countries like Bangladesh the frequency, severity, and nature of the adverse effects of nutritional deficiency of iron are perhaps more problematic. Preterm or low birth weight infants, young children, pregnant women, and women in their reproductive years are more likely to benefit from dietary recommendations to* Lactuca sativa* and* Spinacia oleracea*,* Lablab niger*,* Brassica oleracea,* and* Basella alba* that have high levels of iron to satisfy their greater needs. According to World Health Organization (WHO), 39% of children less than 5 years of age, 48% of 5- to 14-year-old children, and 42% of women in developing countries suffer from anemia and half of these individuals are in iron deficiency. Thus the beneficiary effects of iron can be correlated with significant concentrations present in all vegetable samples.

A very narrow range of concentration (11253–15152 ppm) of cobalt was found between* Basella alba* and* Lablab niger*. Cobalt acts as a cofactor of enzymes that is involved in DNA biosynthesis and metabolism of amino acid [55]. Cobalt is a unique key mineral in Cobalamin or vitamin B12. Cobalt deficiency disease or symptoms are noticeable in vitamin B12 deficiency and pernicious anemia. In humans, cobalt deficiency symptoms include hypothyroidism, goiter, and heart failure. Very little information is available on its concentrations in different kinds of foodstuffs.* Basella alba* and* Lablab niger* are incredibly high in mineral Co. So these kinds of vegetables may prevent diseases like pernicious anemia. RDA per day of Co is 1-2 mg for a normal adult male of 22 years of age established by the Food and Nutrition Board (FNB), National Research Council, 1968 [6]. Cobalt showed quite lower toxicity than many other elements present in the soil of vegetable plots [56]. Cobalt in dietary sources like vegetables and fruits comes from soil contaminated by phosphate fertilizers, sewage sludge, burned fossil fuels, and industries that use or process cobalt containing compounds [57]. Children can be easily affected by coming in contact with high concentration of cobalt found in these vegetable samples because of their smaller body weights.

The amounts of Sr present in both* Basella alba* and* Lactuca sativa* were 1043 and 3015 ppm, respectively. The concentration of Sr in the* Lactuca sativa* grows in polluted areas and may be due to the absorption of the Sr from the polluted soils and water. These types of plants can also be applied to control soil pollution, make the soil safe, and facilitate further cultivation of vegetables in these plots. Strontium has little or no beneficial action. Sr can replace calcium in bone formation and has been used to hasten bone remineralization in diseases like osteoporosis [6]. The presence of Sr in these two vegetable samples might boost the development of collagen and cartilage in joints of human body.

Silicon helps human body to shield against cancers and other severe diseases by neutralizing some heavy metals and scavenging of free radical as well as highly reactive singlet oxygen (^1^O_2_) [28]. Powell et al. reported that fruit and vegetables were the richest sources of Si with substantial quantities present in Kenyan spinach [58]. This study has revealed that* Malabar spinach* (*Basella alba*) and spinach (*Spinacia oleracea*) contain the silicon and may play a vital role in bone formation and also may give protection against cancers. The existence of Si in these vegetables is therefore also helpful for cleaning human teeth, as the plants make the teeth strong and healthy. Only* Basella alba* and* Spinacia oleracea* contained Si in the concentrations of 3271 and 3124 ppm, respectively. Total Diet Study of FDA revealed that only 14% grain products and 8% vegetables were the contributors of Si to the U.S. diet [59]. Si was not detected in* Lactuca sativa*,* Lablab niger,* and* Brassica oleracea*. The Food and Nutrition Board (FNB) has not established a DRI/RDA for silicon since it is not considered essential [37].

Cl is another very indispensable element to human body. For example, Cl joins with Na and K to bring electrical charge when it is dissolved in body fluids that allows working of nerve cells. Its ion is used as an electrolyte, as well as making the hydrochloric acid. It is used by the stomach for digestion of food properly [28]. It also maintains the electrolyte balance in human body. It also helps in absorbing of crucial elements that are required for man's survival. All five vegetables had concentrations of Cl showing difference between 35188–75013 ppm. It also contributes to the maintenance of the electrolyte balance. RDA per day of Cl is 1700–5100 mg for adults and children over 4 years of age [52]. These vegetables may be capable of exhibiting some unique beneficial effects particularly in regulation of the body fluid balance and circulation of ions in the bloodstream.

Both* Spinacia oleracea* and* Lablab niger* analyzed in this work had concentrations of S observed through PIXE were 74442 and 72168 ppm, respectively. But S was not detected in the remaining vegetables. Sulfur is considered to be therapeutically important. Sulfur is widely distributed throughout the body as sulfhydryl groups of cysteine and disulfide linkages in protein from cysteine, for example, insulin [6]. According to National Research Council, 1968, RDA per day of S is 2-3 gm for a normal adult male of 22 years of age [6].* Spinacia oleracea* and* Lablab niger* with their abundant quantities of S may lower blood cholesterol and speed production of carcinogen-destroying enzymes and thus protect against cancer development.

Bromine contents showed the lowest value of approximately 6395 ppm in* Spinacia oleracea* as compared to those in the* Lablab niger* (7485 ppm) and* Brassica oleracea* (6643 ppm). Bromine remained undetected in* Basella alba* and* Lactuca sativa*. Bromine has no known benefit in life processes in plants and animals. So there is no Dietary Reference Intake (DRI)/Recommended Dietary Allowance (RDA) for pregnancy, children, adults, and nursing. It was revealed from the results that all three vegetable samples have higher Br concentration than what these should normally be in vegetables. The uptake of this high concentration of Br by vegetables is influenced not only by plant species and physicochemical characteristics of soil but also by temperature and rain fall which may exert considerable effects.

## 4. Conclusion

PIXE technique was used for the elemental analysis of five different vegetables available in the local market of Savar, Dhaka 1342, Bangladesh. Significant differences were observed during analysis of the concentrations of 18 elements of five different vegetable samples. Considering the order of decreasing concentrations of elements in five different vegetables, it can be concluded that all of them were observed as rich sources of macro elements, that is, K, Ca, Cl, P, and S as well as trace essential elements, that is, Fe, Mn, Cr, and Zn. These vegetables are also free from toxic elements including Cd and Pb. Some elements like Ti, Sr, Ru, and so forth which have no known biological action in human body were found in very low concentrations. This study also revealed that overall distributions of K, Ca, Fe, Cl, P, and Zn in all vegetable samples analyzed are high. The use of fertilizers and metal based pesticides in agriculture may be responsible for these excessive levels of elements which may lead to serious health related complications. The results of this study supply valuable pieces of information about the metal contents in vegetables available in the local market of Bangladesh. Moreover, these results can also be used to investigate the chemical quality of other vegetables available here in Bangladesh and to avoid all the possible health risks associated with their consumption by humans.

## Figures and Tables

**Table 1:** Concentrations (in ppm) acquired for the reference material IAEA-V-10 Hay (powder) and compared certified values.

Element	Measured value	Certified value
Si	Not detected	—
P	2280 ± 70	2300
Cl	Not detected	—
K	21000 ± 45	21000
Ca	21600 ± 60	21600
Ti	Not detected	—
Mn	46.8 ± 1.7	47
Fe	186 ± 11	186
Co	0.11 ± 0.03	0.13
Zn	24 ± 2.3	24
Sr	42 ± 2	40
Br	7.3 ± 0.9	8
Rb	8.1 ± 0.4	7.6
Cr	5.7 ± 0.5	6.5
Ru	Not detected	—
Ni	4 ± 0.2	4
S	Not detected	—
Cu	9.4 ± 0.04	9.5

Values are presented as mean ± SD (*N* = 5). *N* = number of samples analyzed.

**Table 2:** Elemental concentrations (in ppm) of the samples analyzed.

Elements	*Basella alba *	*Spinacia oleracea *	*Lactuca sativa *	*Lablab niger *	*Brassica oleracea *
Si	3271 ± 15.5	3124 ± 26	—	—	—
P	22827 ± 46	65105 ± 63	78342 ± 50	14314 ± 55	87856 ± 45
Cl	75013 ± 51	35935 ± 51	35702 ± 33	35188 ± 64	37523 ± 65
K	91632 ± 90	88021 ± 96	97723 ± 93	92381 ± 85	83412 ± 90
Ca	98652 ± 89	95213 ± 101	86606 ± 102	84615 ± 79	80743 ± 87
Ti	24026 ± 52	33682 ± 70	—	—	—
Mn	1852 ± 16	13222 ± 78	59861 ± 61	39402 ± 52	9018 ± 24.3
Fe	72325 ± 71	83411 ± 87	90525 ± 98	80761 ± 95	72123 ± 82
Co	11253 ± 15	—	—	15152 ± 17	—
Zn	14022 ± 49	26125 ± 38	82467 ± 48	58414 ± 62	15765 ± 31
Sr	1043 ± 22	—	3015 ± 12	—	—
Br	—	6395 ± 19	—	7485 ± 24	6643 ± 16.5
Rb	—	1595 ± 24.54	4426 ± 22	—	—
Cr	—	—	9335 ± 17	—	9112 ± 11
Ru	—	—	6301 ± 20	—	—
Ni	—	—	—	—	1214 ± 13.1
S	—	74442 ± 33.5	—	72168 ± 60	—
Cu	—	—	—	—	1264 ± 14

Values are presented as mean ± SD (*N* = 10) for 18 elements in each vegetable. *N* = number of samples analyzed for each vegetable.
